# A community-engaged approach to examining barriers and facilitators to accessing autism services in Korean immigrant families

**DOI:** 10.1177/13623613211034067

**Published:** 2021-07-21

**Authors:** Vanessa C Fong, Bo Sang Lee, Grace Iarocci

**Affiliations:** 1 Simon Fraser University, Canada; 2 Here and Now Community Society, Canada

**Keywords:** autism spectrum disorders, community engagement, education services, family functioning and support, policy, qualitative research, social services

## Abstract

This study adopted a community-engaged approach to explore Korean parents’ perceptions of barriers and facilitators to accessing autism services in British Columbia, Canada. Understanding the experiences of culturally and linguistically diverse families and their children may help facilitate the development of more inclusive, appropriate, and culturally sensitive services. Semi-structured interviews with 20 Korean parents of autistic children were analyzed using a thematic approach. Barriers and facilitators at the system, provider, and family/cultural level were identified. Barriers at the system level included delays and waitlists for services, and ineffective school policies to address child behavioral challenges. At the provider level, barriers included a lack of qualified professionals, negative attitudes, and lack of guidance navigating services. For family/cultural-related barriers, language and communication difficulties, out-of-pocket costs, and stigma impeded service access. Facilitators at the system level included family-centered care and prioritization of mental health supports. At the provider level, strengths included culturally competent and bilingual professionals. The family/cultural-related facilitators identified were informal support networks, characteristics of the parent, and connections to cultural community organizations. The findings emphasize the need to understand and consider diverse experiences, preferences, and values in the design and provision of autism services for families and their children.

**Lay abstract:**

Perceptions and experiences of culturally and linguistically diverse groups in accessing autism services in Canada are extremely limited. Thus, this study partnered with a community member to explore Korean immigrant parents’ perceptions of barriers and facilitators to accessing autism services in British Columbia, Canada. Interviews were conducted with 20 Korean parents of autistic children. Barriers and facilitators at the system, provider, and family/cultural level were identified. Barriers at the system level included delays and waitlists for services, and ineffective school policies to address child behavioral challenges. At the provider level, barriers included a lack of qualified professionals, negative attitudes, and lack of guidance navigating services. For family/cultural-related barriers, these included language and communication difficulties, out-of-pocket costs, and stigma impeded service access. Facilitators at the system level included family-centered care and prioritization of mental health supports. At the provider level, strengths included culturally competent and bilingual professionals. The family/cultural-related facilitators identified were informal support networks, characteristics of the parent, and connections to cultural community organizations. The findings emphasize the need to understand and consider diverse experiences, preferences, and values in the design and provision of autism services for families and their children.

Families of autistic children report a number of strengths and challenges across the lifespan. Strengths reported include greater closeness among family members, positive relationships between siblings, personal growth, and improved marital relationships (Fong et al., 2021; Mount & Dillon, 2014; Orsmond et al., 2009; Phelps et al., 2009; Rivers & Stoneman, 2003). Families also report a number of challenges raising their autistic child due to the nature and complexity of their child’s social, behavioral, physical, and medical needs (Estes et al., 2013). One factor that has consistently emerged as significant in impacting family functioning is access to disability-related supports and services (Eskow et al., 2019; Fong et al., 2020; Gouin et al., 2016). Autistic children and their families require timely access to a range of services and supports across the lifespan, yet consistently report many challenges in accessing these (Chiri & Warfield, 2012; Vogan et al., 2017).

Barriers to accessing services may be further exacerbated among immigrant families due to language barriers, acculturation stressors, limited support networks, precarious employment and housing, and difficulties navigating unfamiliar education, social and health care systems (Khanlou et al., 2017; Rivard et al., 2019; St Amant et al., 2018). Previous research has revealed differences in how immigrant and non-immigrant parents perceive and understand their child’s disability, their knowledge of developmental milestones, and their recognition of autism symptoms (Magana et al., 2013; Stahmer et al., 2019). These factors are likely to impact a parent’s willingness and approach to seeking services and supports for their child. For example, studies have shown that children of immigrant parents are more likely to be referred for evaluation and receive an Autism Spectrum Disorder (ASD) diagnosis much later than non-immigrant children (Mandell et al., 2002, 2009). Furthermore, research has shown that even after receiving a diagnosis, children of immigrant parents are less likely to access early intervention and other ASD-related services such as school-based supports and speech-language therapy (Habayeb et al., 2020; Khanlou et al., 2017). This is problematic given the emphasis on early diagnosis and intervention and their link to improved developmental outcomes and quality of life in the long-term (Howlin et al., 2009; Volkmar, 2014; Warren et al., 2011). Existing disparities in access to autism services seen in immigrant communities is a growing concern in light of Canada’s increasingly diverse population.

In Canada, accessing autism services is highly complex with eligibility criteria and programs varying considerably across the country with each province developing their own approach. For example, each province across the country will differ in the amount of public and private funding available for early interventions services. In British Columbia (BC) families of children who have received an official diagnosis obtain funding through the provincial Ministry of Children and Family Development (MCFD). Depending on the child’s age, families in BC are allocated a certain amount of money to be used for that year. For children 5 years and under, they are given $22,000 toward eligible intervention services. These services include parent and behavioral interventionist training, equipment, and intervention-related travel expenses. For children between the ages of 6 and 18 years, they are given $6000 per year toward similar services which is intended to supplement standard special education services provided by the child’s school board (MCFD, 2018). Autistic individuals who are over 19 years may receive support through Community Living British Columbia (CLBC), an agency supported by funds from the British Columbia Ministry of Social Development. CLBC services include residential programs (supported, shared, or staffed residential living), employment and skills development programs, caregiver respite services, and individual and family services (mental health, behavioral, support coordination) (CLBC, 2018). To qualify for this program, individuals must be eligible and either meet the *Diagnostic and Statistical Manual of Mental Disorders* (DSM) criteria for Intellectual Disability (ID) or have a diagnosis of ASD that is accompanied with significant adaptive functioning impairments (CLBC, 2018). Consequently, many autistic individuals who are not diagnosed with ID or who do not have significant problems in adaptive functioning are excluded from this program.

Although the service system in BC is based on a family-centered framework designed to empower parents in making decisions regarding the services that best meet their child’s needs, there are also a number of shortcomings to this approach. For example, some have criticized this system for providing poor guidance and information to families regarding available and evidence-based services and interventions. Another concern relates to the substantial out-of-pocket costs that parents remain responsible for covering when they exceed their allocated funding for the year.

These and other issues experienced by families accessing autism services are likely to be compounded in immigrant populations. Promoting equitable access requires a deeper understanding of immigrant families’ experiences accessing services and supports for their child. One of the largest immigrant groups in BC is from Korea. Previous studies indicate that Korean parents may approach and access services differently compared to non-immigrant parents. In collectivist cultures such as Korea, well-being is closely tied to social interdependence, conformity, and social status (Krys et al., 2019). As a result, members from collectivist cultures may experience raising a child with disability differently compared to members from more individualistic cultures. Although stigma may be present in all cultures, there is some research indicating greater stigma around disability in collectivistic cultures due to the association with shame and dishonor (Hwang & Charnley, 2010; Mak & Kwok, 2010). Other studies have observed that Korean caregivers were more likely to view autism as a hereditary disability that jeopardizes marriage prospects for the child (Stahmer et al., 2019). Korean parents were also more likely to be blamed for their child’s disability and accept the belief that their child’s condition was due to poor prenatal practices and parenting behaviors (Cho et al., 2000). In addition, cultural factors related to immigration status and language barriers may contribute to elevated stress in parents (Kim et al., 2020). Together, these cultural elements likely impact service navigation and impede access to autism-related services.

Documented experiences of specific immigrant groups accessing autism-related services in Canada are extremely limited. This coupled with the growing concern in the field for the lack of research participation from individuals from culturally and linguistically diverse backgrounds motivated this study. Understanding the experiences of these families and their children could help expand our knowledge base and would help facilitate the development of more inclusive, appropriate, and culturally sensitive services. In addition, a better understanding of these families and their perceptions of service delivery in BC may help shed light on how best to support these families in ways that are congruent with their culture and values. Thus, this study sought to document the barriers and facilitators Korean immigrant families in BC encounter when accessing autism-related services for their child across the lifespan. We broadly define autism-related services to include diagnostic services, general therapies such as counseling, physiotherapy, occupational therapy, and speech-language therapy, and autism-specific interventions such as Applied Behavior Analysis that are delivered in school, community, and home settings.

## Methods

### Participants

The researchers selected a total of 20 participants using purposive sampling from a list given by a Korean organization providing services, information, and advocacy for families of children with developmental disabilities in the community. This sampling technique was chosen to ensure that parents from diverse income levels, English-speaking abilities, and age ranges were included. Inclusion criteria specified that parents had a child diagnosed with ASD and that their racial/ethnic background was Korean. Participants were not excluded if they did not speak English. All materials were translated in Korean and interviews were conducted in Korean if needed. All parents who were included in the study were immigrants to Canada within the last 20 years (mean (M) = 14.97; standard deviation (SD) = 6.03). Saturation of the themes arising from the interview data guided the final sample of 20 participants.

The majority of our sample were mothers (85%) and the age range was between 39 and 59 years (M = 48.10; SD = 13.02). The most frequent family income reported ranged between $50,000 and $79,999, which is slightly lower than the provincial average income of $84,850 (Province of B. C., 2021). Demographic information for parents and their families are provided in Table 1. The age range of parents’ autistic children was between 5 and 33 years (M = 17.45; SD = 9.20).

**Table 1.** Participant demographic characteristics.

Demographic information	Frequency (%), *N* = 20
Respondent relationship to child with ASD	
Mother	17 (85.0%)
Father	3 (15.0%)
Primary caregiver age	
30–39	2 (10.0%)
40–49	7 (35.0%)
50–59	11 (55.0%)
Marital status	
Married or common law	17 (85.0%)
Divorced or separated	3 (15.0%)
Maternal education	
High school	1 (5.0%)
Professional diploma	1 (5.0%)
Undergraduate degree	11 (55.0%)
Graduate degree	6 (30.0%)
Other	1 (5.0%)
Paternal education	
Undergraduate degree	8 (40.0%)
Graduate degree	9 (45.0%)
Other	2 (10.0%)
Family income	
<$20,000	1 (5.0%)
$21,000–$49,999	4 (20.0%)
$50,000–$79,999	8 (40.0%)
$80,000–$109,999	5 (25.0%)
$140,000–$169,999	1 (5.0%)
>$170,000	1 (5.0%)
Family member most responsible for child with ASD	
Mother	14 (70.0%)
Mother and father	4 (20.0%)
Parents and siblings	1 (5.0%)
Parents, siblings, and other members	1 (5.0%)

ASD: Autism Spectrum Disorder.

### Diagnostic confirmation

Inclusion criteria specified that children included in the study had obtained a standardized clinical diagnosis of ASD from a qualified psychologist, pediatrician, or psychiatrist associated with the provincial government-funded autism assessment network, or through a qualified private clinician. A diagnosis of ASD was guided by the Diagnostic and Statistical Manual of Mental Disorders and confirmed using the Autism Diagnostic Intervention–Revised (ADI-R; Rutter et al., 2008) and the Autism Diagnostic Observation Schedule (ADOS; Lord et al., 1999), both of which are gold standard tools for diagnosing ASD.

Parents also provided information about their child’s intellectual functioning, adaptive functioning, behavioral challenges, and disability severity (Table 2). Since most families did not speak English as their first language, researchers decided to limit the number of surveys required for parents to complete. Instead of having parents complete more lengthy surveys, such as the Vineland Adaptive Behavior Scales–Second Edition Survey Interview (Vineland-II; Sparrow et al., 1984) and the Nisonger Child Behavior Rating Form (NCBRF; Aman et al., 1996), parents reported on these domains by providing ratings that ranged from “Mild” to “Very Severe.”

**Table 2.** Child characteristics.

	Frequency (%), *N* = 20
Intellectual functioning	
Low	1 (5.0%)
Low average	10 (50.0%)
Average	7 (35.0%)
High average	2 (10.0%)
Adaptive functioning	
Low average	10 (50.0%)
Average	8 (40.0%)
High average	1 (5.0%)
Superior	1 (5.0%)
Behavioral problems	
Mild	6 (30.0%)
Moderate	12 (60.0%)
Severe	2 (10.0%)
Child disability severity (*parent-report*)	
Mild	6 (30.0%)
Moderate	7 (35.0%)
Severe	4 (20.0%)

### Interview

A set of open-ended questions comprising our interview guide was developed after consultations with our community partner, self-advocates and their families, and other researchers in the field. The main themes which were the focus of this study explored barriers and facilitators to accessing autism services for their child in BC. Specific questions exploring this topic included “what autism services in BC are you most satisfied with?” and “what challenges or barriers have you experienced accessing autism services in BC?” The interview guide also included a set of prompts and follow-up questions to clarify or obtain additional information. During each interview, detailed notes were taken of the parent’s tone, body language, and areas of emphasis to provide detail and context to their responses. After each interview was completed, a contact summary sheet was completed. This exercise included summarizing the participant’s overall responses to each question, identifying potential themes, and detailing aspects of the interview that were salient or important.

### Procedure

The protocol for this study was reviewed and approved by the Institution’s Research Ethics Board in British Columbia, Canada. Parents who met inclusion criteria for the study (i.e. were of Korean ethnic background and had a child with a formal ASD diagnosis) were contacted by the researcher and those who wished to participate scheduled an interview. The parents completed a demographics form in either English or Korean to accommodate their language needs and preferences. The interviews ranged between 35 and 60 min and were conducted in either English or Korean based on their preference.

The first author conducted and transcribed the interviews done in English and the parent partner conducted the Korean-speaking interviews with participants. Bilingual research assistants fluent in speaking and writing Korean transcribed and translated the interviews. The first step in this process involved transcribing each interview in Korean and the second step required translating the Korean transcripts into English. Each interview was transcribed and translated by two independent research assistants who then compared their final transcripts to verify and ensure accuracy.

### Data analysis

Interview transcripts were coded and analyzed using the NVivo software. The researchers involved in data analysis had background and training in qualitative data analysis and experience in the field of developmental disabilities. A constructivist grounded methodology was used to guide data collection and analysis. This framework guides exploration into how an individual constructs meaning through their experiences and interactions with their environment (Hays & Singh, 2012). This framework is particularly useful for understanding caregiver perceptions of service access because it recognizes subjective interpretations and allows for the analysis of diverse meanings attributed to these experiences. Furthermore, examining the specific contexts, such as cultural norms and practices, that shape and impact a parent’s experience navigating autism-related services and supports is a central aim of this study.

This theoretical orientation involves three stages of coding: initial, focused, and theoretical. Initial coding entailed reading through each transcript several times and highlighting words or sentences which captured the participant’s experience. Fragmenting the data into smaller units allowed for comparisons within and across transcripts to be made. All of the codes generated during the initial coding stage were date and time-stamped and stored in a codebook. This allowed for the ability to evaluate the progression and development of codes into various hierarchical levels. Focused coding, the second stage, required identifying the most significant or frequently mentioned codes and collating these initial codes into higher-order, meaningful units. During this stage, initial codes were re-organized and synthesized into broader categories based on similar properties and characteristics. The final stage of this method, which involved creating theoretical codes, required more in-depth analysis focusing on relationships and connections between the higher-order codes. Memos, which were done for each interview, were weaved through and integrated throughout this stage to support analysis.

### Trustworthiness

In qualitative research, trustworthiness refers to the quality and degree of confidence and trust in the data and the interpretation of findings (Denzin & Lincoln, 2005). Lincoln and Guba (1985) created criteria to evaluate trustworthiness which includes credibility, dependability, and transferability. Table 3 shows the techniques and strategies used to address each criterion.

**Table 3.** Trustworthiness criteria and procedures.

Criterion	Technique	Description
Credibility	Member checking	Interviewers queried and clarified participant responses when needed. Interviewers verified interpretations of data with participants and community consultants.
	Prolonged engagement	This criterion was met though ongoing interaction with the community by co-presenting with members at workshops, participating at community events, and continued research activities.
	Peer debriefing	This technique involved meeting with other researchers and colleagues outside of the study to provide feedback on coding, and theme development.
Dependability	Reflective journaling	This involved keeping a record of all research decisions and justifications, coding definitions and their revisions, and theme development.
	Memos	Memo writing involved taking detailed notes before, during, and after interviews with participants. In particular, documenting non-verbal language, willingness to participate, and any other relevant contextual details.
	Audit trail	Collecting raw data, creating time- and date-stamped memos, and documenting all revisions to coding and theme development were elements included in the audit trail.
Transferability	Thick description	This involved providing rich descriptions, contextual information, and direct quotes from participants exemplifying themes.

### Community involvement

Community engagement was a central focus of this study. Community engagement is when “partners contribute expertise and share decision making and ownership to increase knowledge and understanding of a phenomenon, and integrate that knowledge with interventions, policy advocacy, and social change to improve quality of life for communities and reduce health inequities” (Coombe et al., 2020, p. 553). A community-engaged approach was particularly valuable given this study’s focus on exploring culturally and linguistically diverse families. Adopting this paradigm helps ensure that the research methods used and interpretations of the results are culturally sensitive and accurately portray the lived experiences of the community. Furthermore, adopting this approach has practical benefits for researchers in facilitating the recruitment of hard-to-reach populations.

For this study, we collaborated with a parent of an autistic child throughout all stages of the research. This individual is also the executive director of a non-profit community organization supporting individuals with disabilities and their families. In the initial planning stages, meetings were held to discuss the purpose of the partnership and the various goals for the study. Our partner provided their perspective on the feasibility of the study and the potential impact of the study in addressing their own and their communities’ needs. Their role also included collaborating in developing the research questions, recruiting participants, conducting the interviews, and analyzing and interpreting the results.

## Findings

### Barriers

Three categories of perceived barriers to accessing services were identified in the interviews with parents: those pertaining to (1) the system, (2) service providers, and (3) family and culture. Several subcategories emerged for each type of barrier. The definitions and examples for each theme are outlined in Table 4.

**Table 4.** Summary and description of themes.

Themes	Subthemes	Example codes	Definitions
System-related barriers	Delays and waitlists School policies	“Long waitlists” “Suspension or early dismissal”	Instances where the participant mentions policies, practices, or procedures that negatively impact their child or family
Provider-related barriers	Lack of qualified professionals Negative attitudes Lack of guidance	“Insufficient training” “Lack of trust” “Unaware of services available”	Instances where the participant describes challenges, or negative experiences with health care providers and professionals
Family/cultural-related barriers	Language and communication difficulties Out-of-pocket costs Stigma	“Language barriers” “Ineligible services for funding” “Hiding the diagnosis”	Participant mentions challenges accessing services due to personal, family, or culturally related issues
System-related facilitators	Family-centered care Mental health supports	“Acknowledging expertise of family members” “Mental health counselling”	Participant describes policies, practices, or procedures that have facilitated their access to services and supports.
Provider-related facilitators	Cultural competence Bilingual professionals	“Awareness of cultural values” “Interpreters familiar with autism”	Instances where the participant mentions characteristics of health care providers and professionals that have supported and enabled their access to services
Family/cultural-facilitators	Informal support networks Characteristics of the parent Cultural community organizations	“Emotional support from friends” “Parent willingness to seek information” “Korean church community”	Instances where the participant identifies factors related to their family or culture that have facilitated their access to services

### System-related challenges

Parents often described systemic-related challenges navigating autism services for their child. The most frequently mentioned subcategory was related to the excessive delays and waitlists (*N* = 15), particularly for publicly funded diagnostic services. Waiting periods for accessing publicly funded diagnostic services ranged from several months to 2 years. Some parents resorted to paying out-of-pocket for private diagnostic services despite the high cost as they considered this “buying time”:

Timing is really important, but everything is so slow, and we have to wait for a long time and during that time the situation is getting worse. So, if we can get the right support at the right time this can make a big difference.

The second most frequent type of systemic barrier experienced by parents was related to the lack of procedures or staff training in school settings for supporting students with behavioral challenges (*N* = 7). Several parents reported receiving numerous phone calls throughout the week requiring them to pick up their children from school. This was viewed as highly anxiety-provoking and disruptive to their work, whereby a number of parents had to quit their job to ensure their availability to pick up their child:

At the slightest bad behaviour, it’s just normal for the school to call the parent and ask them to pick the child up. It’s something I’ve also heard from other moms too.

No matter which school—whenever the child’s behaviour is not understood, and behavior problems arise as a result [they will call the parents to pick the child up]. I’m always nervous about it. When a call comes from the school, it might be my personality, but the burden of that is very substantial.

### Provider-related challenges

The second category of barriers reported by parents related to their experiences and interactions with health care professionals and service providers. This could be further divided into three distinct subcategories. The most frequently mentioned obstacle related to difficulties finding qualified professionals to work with their child and the high turn-over rate of behavioral interventionists^1^ (BIs) (*N* = 11):

Some professionals who work with my child don’t have the special knowledge about people with disability. Even at school, the educational assistant does not have this training or special qualification they just get this job and then learn how to do it after.

There is a lot of money wasted and time—my child’s time not mine. I have to teach the BIs every time. I had to learn from them, but I also had to teach them. I need to find the proper person for my son, but it is hard to find someone who is trained to work with people like my son.

While many parents expressed feeling grateful for Canada’s welfare system and recalled positive interactions with health care professionals, there were also a number of parents who reported negative experiences (*N* = 11). During these encounters, parents perceived negative attitudes and a lack of trust in professionals. Mistrust appeared to stem from professionals using unfamiliar or inaccessible language and displaying impatience through their tone of voice and non-verbal language. Some parents expressed feeling discriminated against because they were immigrants particularly when they observed inconsistent service delivery in their community:

When I kind of complained about something, the agency would protect the employee rather than understand a parent’s concern. When we were in a meeting, they said this is the last time we’re going to do this for you as if they were doing me a favor. They said it in that tone.

I’m not sure whether this is because we are Korean or not, I don’t know. But that’s what I have seen within my community here.

Another barrier accessing services experienced by parents included a lack of guidance provided by professionals and feeling the need “to figure it out on their own” (*N* = 9). Parents articulated the need for more guidance and information upon receiving a diagnosis. Many parents mentioned the need for information beyond what services were available, with a more individualized approach on how each service may be beneficial for their child based on their needs:

During the initial diagnosis stage, the psychologist gave me this paper that said “okay your son is on the spectrum” and that’s it. I felt that she threw me into this pit without any guidance and said you need to climb up by yourself, you’re on your own now. You’re in this deep pit called autism and you got to figure it out yourself and it’s going to be daunting, and I cannot help you because I’m busy.

When you’re a mother that doesn’t know much, specific instructions need to be given about what can be received from which people and which institutions, so they can spend the money accordingly. These kinds of guidelines need to exist, but it doesn’t. Since there are no guidelines, parents struggle, and we don’t know where to use this money.

### Family- and cultural-related challenges

Family- and cultural-related obstacles were further divided into three distinct subcategories. The most frequently reported barrier was related to language and communication difficulties (*N* = 14). Limited English proficiency impacted parents’ ability to seek and understand information available, and negatively impacted their willingness to engage with support services and communicate their desires and needs to professionals. Parents also expressed feeling uncomfortable and shy communicating with professionals and disclosing health information and felt more at ease bringing along a friend or family member to appointments. Some parents reported perceiving themselves as passive recipients who needed to learn to adjust their communication styles and conform to society:

Some families have lots of language barriers. They can’t demand, they can’t express themselves or what their son’s or daughter’s wants or needs are. I often see individuals who are very, very isolated.

You have to have this gentle, smiley face. It’s very different from Korea. Because in Korea if you have a louder voice and you use harsh words then you get what you want. But it’s very different here. If you do that here, then they will think that you are very abusive and that it’s not polite. Here you have to be very strong but in a very subtle, gentle way. You have to learn that kind of custom.

Parents reported substantial out-of-pocket costs to fund essential services not covered publicly (*N* = 12). It was common for parents, typically mothers, to quit their jobs to manage and coordinate various services and supports for their child. This was extremely time-consuming especially given the high turn-over rate for BIs which involved constant hiring and training team members:

It’s just not enough—the funds. I know we are getting some but most of the families I know use out-of-pocket expenses in order for them to maintain a good quality therapy. And me too, I spend a fortune on out-of-pocket expenses for my son’s therapy in order to have a lot of intensive therapy, and we’re desperate right? All families are desperate because we think this is early intervention, we think if we miss this time they will make progress slower. We believe that this is the critical time, so we spend a fortune and it’s not even enough and it drops drastically after 6 years old.

Another barrier frequently mentioned by parents was related to stigma (*N* = 10). A number of caregivers attributed their own reluctance to obtain a diagnosis as due to fear of stigmatizing their child and family. These caregivers discussed the negative view of disability in Korean culture as reasons for keeping their child’s diagnosis a secret from grandparents, extended family members, and friends:

I cannot even talk to my parents still. I’m having such a hard time talking with my parents. They’re still denying it. Certain things I cannot even talk to my wife about, but you know the church helps me. I pray for stuff that I cannot talk to anybody about.

He [husband] was more of a typical kind of “Asian Korean man” who couldn’t really accept his kid’s challenges. He tried hard but sometimes that made my kids’ life a little bit difficult.

People are not open about having a disability in general in Korea or Asia. They want to hide it, they’re not going to tell people “oh my son has disability.” They will just keep them at home, they won’t go outside, they don’t tell people. They are just ashamed, even though there is nothing to be ashamed of.

### Facilitators

Three categories of perceived facilitators to accessing services were identified in the interviews with parents: those pertaining to (1) system, (2) services providers, and (3) family and culture. Several subcategories emerged for each type of facilitator. The definitions and examples for each theme are outlined in Table 4.

### System-related facilitators

Parents most often identified the importance of family-centered care and having control over how to spend their child’s funding as a strength (*N* = 9). In particular, parents were satisfied with the At Home Program^2^ which provides medical benefits and respite for families with severe disability or complex health care needs. Despite strict eligibility criteria and long waitlists, families who were able to access this program found it very helpful:

The funding is really important, I’m really grateful for that. Through ABA therapy, although there are still some difficulties, [our daughter] doesn’t negatively affect our family’s quality of life greatly. Right now, I don’t feel like the family is having a hard time because of her or that our lives are made difficult because of her. When she grows up there might be some changes, but for now she’s giving us joy in life. I don’t think there has been anything too difficult yet.

So now he’s out of the ministry services but the most impressive services he was getting was the At Home Program. It delivers the medical benefits and the respite benefits and it’s not really easy to be eligible for the services but once you’ve passed eligibility assessment it can be a great help. So I liked this service the most.

The availability of mental health supports and counseling was also seen as another important service identified by parents (*N* = 8). Parents reported the need to prioritize mental health supports for their autistic child especially during the diagnosis and transition periods. Parents also highlighted the need for both individual and family counseling to support and help family members understand and cope with difficulties encountered in their daily lives:

He has support for mental health and began to have this around age 16, around puberty. It is very hard to get in BC and it requires referral from both the pediatrician and ministry. It was very hard to get but I think it is a very useful service for kids, teens, teenagers.

I really worked hard and eventually I got burnt out and I got really depressed for a while because I didn’t take care of myself and I didn’t have any breaks. Counselling was helpful, talking to somebody about my concerns and being heard. I had this therapist, and she would come and visit me at my home and listen to all my concerns related to my husband, son, or whatever. But usually, it was related to my son’s diagnosis because that was affecting me.

### Provider-related facilitators

Parents mentioned the importance of cultural competence and working alongside professionals who were patient, empathetic, and understanding (*N* = 8). Caregivers appreciated when providers went the extra mile to provide information and advocate on behalf of the family. In addition, consistent and effective communication with providers was viewed as a strength for many parents:

Our social worker was a retired police officer. She was really into fighting for your rights and doing the right thing. And she was very helpful. And she knows the whole story and she goes “you went through so much that you didn’t have to.” And she was really helpful, and she gave me lots of information that I didn’t even ask for.

When I can talk to the teachers more comfortably and there is good communication we can plan together in more detail and we can create a more specific plan that will help my child in the future.

Korean parents believed that service access was aided by interactions with bilingual professionals and having access to interpreters with specific knowledge of the autism service system (*N* = 8). Parents expressed the need for more culturally diverse professionals to be involved in the field and the need to employ more Korean professionals in mainstream healthcare fields in order to improve communication between service providers and service users:

I think we could get a lot of help if we had people working who were proficient in English and Korean. Our family is satisfied now because of cultural community organizations that have been developed, we are getting a lot more help than before. Our child is still doing speech therapy today, but I often think about how good it would be if the person doing the speech therapy was a Korean person. So, when I have conversations with the teacher, I’m able to say everything I want to. If Korean people could work with our children in education, schools, and service agencies it would help a lot.

### Family- and cultural-related facilitators

Caregivers frequently identified supports from within their own family and broader social support networks as facilitating their access to services (*N* = 13). Participants shared the importance of having their family and/or friends involved in the initial diagnostic process for emotional support and to aid in translating. Specifically, parents found help with babysitting, moral support and advice, and transportation particularly useful:

I think that unnie [family friend] was the one that recommended getting a diagnosis and we went together and she translated.

Without information and without any connections it’s very hard for families. It’s not just about obtaining information it’s about making connections in order to enjoy and live life.

For me it was emotionally the most difficult thing in my life. Around the time of the diagnosis, I had a hard time and was unhappy. We felt our family was being isolated from people, even those we had known in the past. And I instantly noticed that the path of our life would be different from theirs and we didn’t have much in common to say about things like how to raise a child and how to get them to succeed academically. Their path was far different from ours. The feeling of isolation was very difficult.

Parents also attributed their own expertise and information-seeking (e.g. parent workshops, searching online, connecting with other families, and reading articles) as facilitating their access to supports and services (*N* = 9). Parents believed that their persistence, courage, and willingness to “fight” for resources and advocate for their child was crucial. In addition, parents consistently mentioned the importance of learning English in facilitating this process:

You have to be brave; the people will understand you no matter how bad your English is and they will try to get you help. That’s why translators are pretty important because there’s a lot of families that have issues because they cannot communicate well. But at the same time, you have to be strong even though your English is not good you have to be brave.

As immigrants I believe it is crucial to learn the language in spoken and written form. Both are important in order to make a proper request to the government. We need to learn to understand the system and how to arrange or reach the supports in BC. If you don’t know the system, there is less capability to access services. Also, I believe that it is crucial to be a strong advocate for your kid because if you don’t, nobody will.

Many parents emphasized the importance of cultural community organizations in facilitating access to formal and informal supports and services (*N* = 8). For many families, these organizations appeared to address social, cultural, and language barriers typically overlooked by mainstream services. Some parents reported that as a minority group, the majority of available services tend to conflict with Korean culture. For example, independence is highly valued in North American culture whereas traditional Korean parents prefer having their child in their own care and homes as long as possible. These community organizations also helped parents by providing translated information, practical advice, emotional support, and guidance allowing parents to feel more prepared and in a better position to advocate for their child:

There are many families in this organization that are in the same situation, so we try to get a lot of help from these families. They tell us where to go, what to do, whom to contact, and all sorts of things.

The government should support these kinds of community organizations because by supporting these organizations the families who receive these benefits can contribute and give back to other families.

## Discussion

### Barriers

Similar to non-immigrant families, Korean immigrant parents of autistic children report delays when accessing diagnostic and complimentary services. However, it is likely that these challenges may be exacerbated in Korean families who are newly immigrated, single parents, have a language barrier, are cut-off from previously held support systems, and are unfamiliar with the healthcare system and services available. Although public diagnostic services are funded by the government, there are long waitlists, in some cases over 2 years, delaying access to early interventions and much needed supports. The impact of these issues is likely compounded in marginalized and low-income communities. Providing families that are below a certain income threshold additional funding for private diagnostic services may help reduce disparities.

A lack of procedures in schools and training of school staff to support children with behavioral problems was seen as another systemic barrier that has important implications. This is consistent with findings by Kim et al. (2020), where Korean parents also perceived a lack of confidence in the school’s ability to support their children and address their needs. In this study, it was specifically the practice of dismissing students in the middle of the day that raised the most concern for parents. Parents identified the causes of early dismissal in schools as due to poor understanding of the causes of their child’s behavioral challenges and failure of the school to implement appropriate supports to mitigate these problems. Consequently, these students and their parents are more likely to experience segregation, isolation, parent job loss, and missed educational opportunities. Policies and practices that support students with challenging behaviors and diverse learning styles in positive and inclusive ways are urgently needed.

In terms of provider-related challenges, negative attitudes, perceived discrimination, miscommunication, and a lack of understanding and empathy from service providers appeared to fuel mistrust and hinder parents from seeking services and supports. Similar findings were obtained by Choi et al. (2017). Their results indicated that Korean parents living in New Zealand also perceived a lack of trust toward professionals and service providers working with their children. This highlights the need for service providers to prioritize building relationships and fostering trust with Korean parents. This could be achieved by listening to and understanding the cultural values and expectations of these families. Approaches aimed at building and maintaining relationships may include learning about a family’s cultural background, key words and phrases in their language, family values, and being mindful of non-verbal communication. Relevant training may also highlight the importance of culturally humble and responsive practices (Ratto et al., 2017; Wright, 2019). Indeed, the current results are consistent with evidence demonstrating that unaddressed cultural differences can negatively affect family engagement and adherence to evidence-based interventions (DuBay et al., 2018).

Family factors such as language and communication barriers, and insufficient funding for services were barriers that limited parents’ access to services and supports. This is consistent with previous research conducted in other countries examining barriers to service access in Korean families (Cho et al., 2000; Cho & Gannotti, 2005; Kim et al., 2020; Y.-J. Lee & Park, 2016). In this study, the loss of employment for parents who quit their jobs to take full responsibility for their child’s care created further economic burden in addition to isolating parents from their former social networks. It was also not uncommon for parents to report feeling isolated and a sense of loss due to sacrificing their careers and hobbies to support their children.

Another barrier reported by parents related to cultural factors such as stigma. In line with previous studies (Burkett et al., 2015; Lopez et al., 2019), caregivers who experienced stigma were more likely to report delays in accessing diagnostic and autism-related services for their children. This finding emphasizes that service delivery and implementation without an understanding of Korean culture may impede access for Korean families. Parents expressed feelings of shame and frustration when taking their child out in public or sharing their child’s diagnosis with family and friends who were unfamiliar with autism because of the reactions and judgments they experienced. Efforts to increase public awareness of autism and other developmental disabilities in the community may help improve understanding, empathy, and acceptance. In addition, this may help foster more inclusive attitudes toward autism as a public responsibility rather than solely a personal or familial responsibility. Aligning with previous recommendations (Kang-yi et al., 2018), this can be facilitated by engaging church or other trusted community leaders who can help address cultural beliefs and stigma. This may enable Korean parents to feel less reluctant to go to public places and may lead to more opportunities for their children and other family members to explore and experience life more fully.

### Facilitators

Despite challenges related to immigrating to a new country and accessing autism-related services, Korean parents are highly resilient and resourceful when it comes to caring for their autistic child. They are able to draw strength from various areas and appreciate the individual talents and sense of fulfillment and joy their child brings to their families. This finding is consistent with previous research revealing positive aspects of caregiving and improvements in family functioning for Korean parents raising autistic children (Cho et al., 2000; Fong et al., 2021).

System-related facilitators such as provision of services using a family-centered approach were viewed as a strength. In addition, service delivery systems that prioritize mental health for both the autistic individual and family were seen as a facilitator. Korean mothers have a strong sense of responsibility and obligation and primarily assume the caretaking role in their households (Grinker & Cho, 2013; Park, 2007). Although they are highly dedicated and committed to this role, they are at a high risk for caregiver burnout and social isolation (Kim et al., 2020; K.-S. Lee & Jung, 2005). A number of parents highlighted this as a major concern and expressed that they wish they had more mental health supports and prioritized self-care. In addition to mental health supports, parents identified that respite played a crucial role in helping them alleviate stress and giving them time to engage in self-care.

Cultural competence in service providers and professionals working with families was perceived as a facilitator in accessing services. Members of Asian cultures may be highly sensitive and attuned to non-verbal and indirect language (Claramita & Susilo, 2014). For example, a number of parents reported feeling covert racism and discrimination in their interactions with service providers. It is crucial for professionals to gain awareness of different cultural practices, beliefs, and value systems and receive cultural sensitivity training. Similar to recommendations from previous studies (Cho & Gannotti, 2005; Kim et al., 2020), the current findings indicate the importance of having translators present who were both bilingual and were familiar with the autism service system. It was crucial that parents felt they could trust the translator to accurately convey their needs and requests and have their best interest in mind. They found it particularly helpful accessing services when professionals and interpreters working with them had knowledge of the system, a strong relationship with the family, and advocated on their behalf.

Throughout many of the interviews, parents consistently identified the importance of cultural community organizations in their daily lives. Specifically, parents mentioned the need for Korean organizations that are staffed by Korean professionals as they may be more effective and better equipped to address complex cultural dynamics that ultimately impact service access. Indeed, previous research has echoed this need for Korean organizations run by members that belong to this community that are familiar with the language and culturally appropriate services for children are families (Choi et al., 2017). Parents anticipated that these organizations would help provide more culturally appropriate services for the children and their families. Importantly, these organizations can also create employment opportunities for Korean parents contributing to greater financial independence and better overall outcomes for their children. Together, these findings suggest that governments should leverage and build capacity in these community-based organizations that serve diverse populations given their effectiveness in terms of community outreach and providing service navigation support.

Findings also highlight the importance of building the infrastructure for service providers to assist parents with service navigation. Support with service navigation is a clear need for many Korean families. Professionals must be dedicated to assisting families in navigating complex service systems and equipped in their role to provide culturally sensitive services and supports. Newly immigrated families require culturally adapted services which may be mediated by navigators comprised of parents from similar backgrounds and experiences. These navigators should provide check-ins as needed, follow-ups, and provide referrals and recommendations to allow for more supportive guidance to help families fully engage with the service delivery system. This is especially important when a range of comprehensive services are offered through distinct sectors including health, education, child and youth, and community services as is the case in BC. Better integrated and coordinated services across various sectors are urgently needed to help immigrant families better access and benefit from autism-related services and supports.

Another contribution of this study relates to the implementation of a community-engaged approach in exploring service navigation in a culturally and linguistically diverse community. The majority of research studies on service access in immigrant populations have relied heavily upon traditional empirical approaches where data are collected *from* the community and programs are designed *for* the community rather than in partnership with individuals with lived experience. There are even fewer studies that engage partners as co-researchers beyond the recruitment stage. This study has demonstrated the feasibility and advantages of engaging community members throughout the analysis stages of the research. Specifically, the partner’s contributions to the analysis stage, to name a few, include providing a diverse perspective to the research, contextualizing findings, highlighting strengths, and ensuring respect and dignity for cultural identities and values.

## Limitations

There are a number of limitations that warrant caution when interpreting the findings. The small sample size and the majority of respondents being mothers may limit the generalizability of findings. In addition, the recruitment of Korean parents who were contacted from a local, non-profit organization in the community may not represent the views and experiences of parents who may be less connected with their communities.

## Conclusion

This study revealed barriers and facilitators at the system, provider, and family/cultural level. Barriers at the system level included delays and waitlists for services, and ineffective school policies to address child behavioral challenges. At the provider level, barriers included a lack of qualified professionals, negative attitudes, and a lack of guidance navigating services. For family and cultural-related barriers, it included language and communication difficulties, out-of-pocket costs, and stigma impeded service access for families. Facilitators at the system level included family-centered care and prioritization of mental health supports. At the provider level, strengths included culturally competent and bilingual professionals. The family and cultural-related facilitators identified were informal support networks, characteristics of the parent (e.g. resourcefulness), and connections to cultural community organizations. The findings highlight the need to better understand and consider the experiences, preferences, and values of culturally diverse communities in the design and provision of services and supports.

## Notes

1. BIs work one-on-one with your child on goals developed by professionals working with the child (Autism Community Training [ACT], 2021). They are supervised and trained by a qualified Behavior Consultant who specializes in designing and implementing Applied Behavior Analysis (ABA) intervention programs.

2. The At Home Program is a government-funded program aimed to support children and youth with severe disability or complex health care needs (Ministry of Children and Family Development, 2018). To qualify for either respite or medical benefits offered by the program, children must be assessed as dependent in three out of four areas of daily living (eating, dressing, toileting, and washing). Those dependent in all four areas are eligible for both benefits.
